# In memoriam: Joep Lange MD, PhD

**DOI:** 10.7448/IAS.17.1.19401

**Published:** 2014-09-08

**Authors:** Chris Beyrer, Stefano Vella, David A. Cooper

**Affiliations:** 1 Department of Epidemiology, Johns Hopkins University, Baltimore, MD, USA; 2 Department of Therapeutic Research and Medicines Evaluation, Istituto Superiore di Sanità, Rome, Italy; 3 The Kirby Institute, University of New South Wales, Sydney, Australia

Professor Joep Lange lived a life of profound consequence. One of the true leaders in our field, his life and work played fundamental roles in shaping and directing the global response to HIV/AIDS – by any measure one of the great human, medical and public health movements of our time. Joep was a past president of the International AIDS Society. His killing, with his beloved partner and muse Jacqueline van Tongeren, in the MH 17 attack of July 17, 2014, will always be remembered by our community as one of our darkest days. He and Jacqueline were en route to the 20th International AIDS Conference, and were deeply, actively engaged in HIV research, education, the movement for universal access and in the emerging interactions of HIV/AIDS and global health. Efforts are underway to memorialize all of those lost on MH 17. Yet, it is only fitting that we, the community of those engaged in global AIDS, pause here to honour Joep Lange.

Joep Lange's medical and scientific career traces an arc of the global response to AIDS. He finished medical training in his native Netherlands just as the epidemic began to break. His early work in the 1980s and 1990s focused on HIV pathogenesis, on the mechanisms of immune compromise seen in HIV infection and in the ravages of, the then largely untreatable, clinical AIDS. This early work on HIV pathogenesis and disease progression helped shape Joep's truly visionary understanding of how HIV kills and how anti-viral therapies would likely need to interrupt these mechanisms. He was among the first to propose that multiple agents would be needed to disrupt the virus at multiple points in its replication cycle, and among the first to suggest that anti-viral agents might be used to interrupt maternal–infant transmission. This key insight of the preventive potential of antiretroviral drugs is arguably the seed crystal of the momentous changes underway in HIV epidemic control – the strategic use of antiretroviral drugs (ARVs) for prevention and treatment, spanning early treatment as prevention to pre-exposure prophylaxis. He published with his Dutch colleagues an early and enormously influential paper in the *Journal of Virology* in 1992 on cellular reservoirs, which provided still relevant insights into HIV persistence [1] – the fundamental challenge to the next great scientific hurdle before us, that is, HIV cure.

By 1992, Joep had joined WHO as Chief of the Clinical Research and Product Development in what was then the Global Programme on AIDS. His career as a catalyst of HIV research in developing countries had begun. In 1996, he co-founded the flourishing HIV–NAT research collaboration with Drs Praphan Phanuphak and David Cooper, bringing together Dutch, Thai and Australian researchers and clinicians to build a research facility in Bangkok, Thailand, then and now, one of the epicentres of HIV in Asia. At that time, many thought that doing clinical research on antiretroviral therapy in the developing world was impossible, given the substantive barriers of minimal impact, monitoring of response and toxicity and cost. The prevailing opinion was that perhaps one could study HIV-associated pneumonia and tuberculosis, but it was Joep's belief that setting up research partnerships with academic advocates such as Praphan Phanuphak, who were also passionate and knew the local landscape well, would succeed. Succeed it did and from a staff of three, one Dutch physician, one Thai nurse manager and one Thai laboratory scientist in 1996, HIV–NAT developed into a sophisticated, world-class, effective and efficient clinical research consortium answering critical questions in the application of antiretroviral therapy to the developing world.

His leadership of the IAS, too, was exemplary. He was instrumental in the IAS decision to move the international conferences to resource-limited settings, together with Mark Wainberg and Stefano Vella, despite the many who were sceptical about the feasibility of such a shift [2]. The success of the 2000 Conference in Durban, and the subsequent one in Bangkok, is now considered a milestone in the battle towards universal access to treatment and care for all persons living with HIV.

In the past decade, as HIV treatment finally became widely available to the millions who needed it in developing countries, Joep's forward thinking led him to a greater focus on health systems and on the integration of HIV services. He helped find the Amsterdam Institute for Global Health and Development, and was its Director at the time of his death. In 2007, along with international colleagues and African scientists, Joep launched the *International Workshop on HIV Treatment, Pathogenesis and Prevention Research in Resource-poor Settings (INTEREST)*, an international annual meeting held in Africa, which focuses on issues of particular relevance to the African continent.

The IAS will never forget Joep Lange for his leadership, his vision, his many scientific contributions, but most of all for his friendship. For those who had the privilege to know him, Joep was a man of great humour, passion for life and a gift for friendship. Perhaps, the best way for us all to honour him is to try and live our lives, and dedicate to our work, with Joep's unflagging energy, intolerance for delay and attention to the future. He was always a man ahead of the curve.

## References

[1] Schuitemaker H, Koot M, Kootstra NA, Dercksen MW, de Goede RE, van Steenwijk RP (1992). Biological phenotype of human immunodeficiency virus type 1 clones at different stages of infection: progression of disease is associated with a shift from monocytotropic to T-cell-tropic virus population. J Virol.

[2] Hogg R, Cahn P, Katabira E, Lange J, Samuel NM, O'Shaughnessy M (2002). Time to act: global apathy towards HIV/AIDS is a crime against humanity. Lancet.

